# A Survey of Farm Management Practices Relating to the Risk Factors, Prevalence, and Causes of Lamb Mortality in Ireland

**DOI:** 10.3390/ani12010030

**Published:** 2021-12-23

**Authors:** Dwayne Shiels, Jason Loughrey, Cathy M. Dwyer, Kevin Hanrahan, John F. Mee, Timothy W. J. Keady

**Affiliations:** 1 Teagasc, Animal & Grassland Research & Innovation Centre, Mellows Campus, Athenry, H65 R718 Co. Galway, Ireland; Tim.Keady@teagasc.ie; 2 Animal & Veterinary Sciences, SRUC, Roslin Institute Building, Easter Bush Campus, Midlothian EH25 9RG, UK; cathy.dwyer@sruc.ac.uk; 3 Teagasc, Rural Economy & Development Centre, Mellows Campus, Athenry, H65 R718 Co. Galway, Ireland; jason.loughrey@teagasc.ie (J.L.); kevin.hanrahan@teagasc.ie (K.H.); 4 Animal and Bioscience Research Department, Teagasc, Moorepark Research Centre, Fermoy, P61 C997 Cork, Ireland; john.mee@teagasc.ie

**Keywords:** ewe productivity, gross margin, questionnaire, hygiene, colostrum

## Abstract

**Simple Summary:**

A reduction in lamb mortality would benefit farmers both economically and ethically. The major causes of lamb mortality are similar worldwide. Targeting the specific causes can result in reduced lamb mortality. This involves identifying underlying factors associated with lamb mortality and subsequently recommending changes to management practices. The objective of this study was to investigate the risk factors associated with lamb mortality on Irish sheep farms to provide a greater understanding of the necessary management practices required to reduce lamb mortality. This was achieved by identifying relationships between on-farm practices and risk factors of lamb mortality associated with these practices. Predators, lamb birth weight, and diseases were perceived by farmers to be the main causes of lamb mortality. Individual lambing pens were used on most sheep farms but were not cleaned and/or disinfected on 26% of them. Lamb mortality tended to be lower on farms that used best-known practices. Full-time farmers that used hospital and individual pens had a higher gross margin (€18/ewe). Management systems affect both lamb mortality and flock gross margin. Every 1% decrease in average lamb mortality across Irish flocks is worth ~€3 million annually to the Irish sheep sector.

**Abstract:**

Lamb mortality is a key factor influencing ewe productivity and profitability. The current study investigated risk factors associated with and management practices implemented on sheep farms to reduce lamb mortality. A survey consisting of 13 multiple-part questions (57 separate questions) was administered to all sheep farmers participating in the Teagasc National Farm Survey, representative of the Irish national population of sheep farms. A total of 60% of respondents identify mating or lambing date, and this practice tended to be associated with reduced lamb mortality (1.2%, *p* = 0.08). Individual lambing pens were used by 88% of farmers, but 26% did not clean or disinfect them. A total of 79% and 9.5% of farmers applied iodine to all lambs’ navels and administered antibiotics to all lambs to treat and/or prevent diseases, respectively. Most farmers vaccinated their ewes (86%) and lambs (79%) against clostridial diseases and/or pasteurellosis; 13% vaccinated against abortion agents. Lamb mortality tended to be lower (Kruskal–Wallis (KW) = 2.749; *p* = 0.09) on farms that used stomach tubing, heat box, iodine, hospital, and individual pens compared with farms that do not implement all those practices. Predators, lamb birth weight, and diseases were perceived by respondents to be the three main causes of live-born lamb mortality. The gross margin is significantly higher on lowland farms by €37 per ewe compared with hill farms (Kruskal–Wallis (KW) = 4.056; *p* < 0.001). The combination of full-time farming and the use of hospital and individual pens improved gross margin (€18/ewe, *p* = 0.028). It is concluded that on-farm management practices affect both lamb mortality and flock gross margin.

## 1. Introduction

The number of lambs reared per ewe joined is a key determinant of productivity, and profitability, in mid-season prime-lamb production and meat sheep systems worldwide [1,2,3,4,5]. Ewe productivity targets vary depending on the system of production within and between countries. In Ireland, the mean ewe productivity on lowland grass-based and hill systems of sheep production is 1.33, and 0.91 lambs reared per ewe joined, respectively [6], and has been similar since the 1960s [3,7,8]. Previous studies at the Teagasc Research Centre, Athenry, Ireland, have shown that it is possible to consistently rear 1.9 lambs per ewe joined on lowland grass-based systems [3,9].

Lamb survival is a significant factor affecting ewe productivity [4,10,11]. Worldwide, estimates of mean lamb mortality (lambs that are born alive and die pre weaning) range from 8% to 30% [3,12,13,14,15,16], and high levels of lamb mortality are a welfare concern [11,17]. Lamb mortality is a multifactorial problem [18]. While it is known that the major causes of lamb mortality are similar worldwide [19], the prevalence of these causes can vary across management systems [18,19,20,21,22]. Prime-lamb production in Ireland is from grass-based systems with ewes predominantly outdoors. Some are housed during late pregnancy but then turned out to pasture with their lambs within days of giving birth. Similar systems of lamb production are practiced in other countries with temperate climates, including the U.K., New Zealand, and regions of continental Europe, South and North America. For ewes lambing indoors, the main causes of lamb mortality are infection and dystocia [15,23], while for ewes lambing outdoors, the main causes of lamb mortality are exposure/hypothermia and predation [24,25,26]. A significant reduction in lamb mortality can only be achieved through identifying the underlying causes, many of which are relevant across different systems of production, and targeting them specifically [16,27]. Implementing effective management practices associated with specific causes of lamb mortality, from before joining ewes (with the ram) to the immediate and subsequent days post parturition provides major opportunities to improve lamb survivability [15,17]. A reduction of 3 percentage points in lamb mortality would be worth approximately €10 million to the lowland sheep sector in Ireland [28] and is the equivalent to 100,000 more living lambs each year. Therefore, it is necessary to know what management practices are implemented on commercial farms and their potential effects on lamb mortality to enable advanced knowledge and management practice transfer aimed specifically at reducing lamb mortality, thus improving ewe productivity.

Survey analysis is a highly efficient method of accumulating large volumes of data rapidly and at a relatively low cost [29]. The Teagasc National Farm Survey (TNFS) has collected detailed data on farm activities, resources, gross output, input costs, and income, as well as other socio-demographic data from a statistically representative random sample of Irish farms since 1972. The TNFS is part of the EU-wide Farm Accountancy Data Network (FADN), the only source of microeconomic data collected from representative samples of the agricultural holdings in each EU member state (Council Regulation 217/2009 [30]. Each year the TNFS conducts a supplementary survey that is used to collect additional information from TNFS respondents. This information can be combined with the wide set of socio-demographic and economic data collected as part of the core TNFS. The information in the supplementary survey is used to examine research questions of importance to producers and those operating similar-type systems worldwide.

The objective of the current study was to investigate farmers’ perceptions of the risk factors associated with lamb mortality on commercial sheep farms, identify management practices implemented on farms to reduce lamb mortality, and evaluate relationships between on-farm practices, gross margins, and the risk factors associated with lamb mortality.

## 2. Materials and Methods

### 2.1. Overview

In 2017, a supplementary survey (additional in Supplementary Materials) was undertaken in addition to the core TNFS. The TNFS sample of approximately 1000 farms represents a farming population of approximately 92,600 farms [6]. The survey was designed to create a profile of on-farm practices that carried both high and low risks for lamb mortality, to collate farmer opinions of the causes of lamb mortality on their farms, and to identify differences in gross margin and weaning rate between adopters and non-adopters of certain management practices.

Data on lamb mortality on each farm were determined by the number of live lambs born minus the number of lambs reared (at approximately 100 days)/100. The data on the number of live lambs born and the number of lambs reared are collected for all farms within the core TNFS. The effects on gross margin per hectare were investigated by dividing farmers into adopters and non-adopters of each practice surveyed.

### 2.2. Farm Selection and Survey Method

The core TNFS survey and the supplementary risk factors survey were completed on paper, on farm, by the respondents with the assistance of trained survey recorders from the TNFS. Only farmers with a sheep enterprise consisting of 20 or more breeding ewes and in excess of €8000 euro standard output [6] participated in the survey. Farms participating in the TNFS are selected with the assistance of the Ireland Central Statistics Office (CSO) to be statistically representative of the Irish farm population. One hundred and eighty-three respondents completed the survey (97% response rate).

The CSO conducts a census of agriculture every 10 years to record the population of farms and the structure of farming in Ireland. Farm structure surveys (FSS) are conducted by the CSO in the intervening periods to produce estimates of the total farm population. These two sources of information on the size and structure of the Irish farm population are used to create panels of farms from which participants in the TNFS are selected. These panels are structured by the CSO to ensure that the farms canvassed to participate in the TNFS are representative of the population of Irish farms with in excess of €8000 standard output.

A proportion of the farmers surveyed each year by the TNFS are replaced annually. Potential recruits to the NFS are approached by a trained survey recorder who informs them what is involved, and they are given the option to agree to be part of the survey or to opt out. Where a farmer declines to participate in the TNFS, another farmer from the list of potential participants prepared by the CSO would be approached. This process continues until sufficient farmer participants across the TNFS sample strata required are recruited. Farmer participation in the TNFS and all FADN member surveys is voluntary. There is no monetary reward to the participating farms in the survey.

Every farm that participated in the current survey was assigned a population weighting factor provided by the CSO. This allows weighted averages across the records of surveyed farms to be calculated that are statistically representative of the national population of sheep farms.

### 2.3. Survey Design

A questionnaire consisting of 13 multiple-part questions (57 separate questions) was developed to create a profile of on-farm practices that carried high and low risks for lamb mortality (Table 1). The questions were predominantly closed (*n* = 53), with some open-ended questions included (*n* = 4). The survey was designed with the specific aim of obtaining farmer responses in the most efficient and easily understood manner. Attention was given to ensure questions were structured/phrased to avoid leading the respondent to a given choice and to avoid bias. Prior to being circulated in the TNFS, the questionnaire was validated by piloting with 10 Teagasc advisory, technical, and farm recorder staff who provided feedback. All feedback and proposed amendments from the pilot study were incorporated prior to completion of the final draft. The final draft of the questionnaire was presented to the TNFS recorders, who were trained and briefed on each question. The supplementary survey took approximately 15 min to complete. Farmers were asked to provide details about their farm (area, livestock numbers) and sheep system (lowland, hill), and lambing practices (lambing indoors or outdoors).

### 2.4. Statistical Analysis

The data were cleaned and screened for anomalies; any unanswered questions were labeled ‘non-response’ (*n* = 34). Of the 183 responses, 177 (97%) were deemed usable for the analysis of risk factors, while 172 (94%) were deemed usable in comparing outcomes for adopters relative to non-adopters of particular management practices. Six survey responses were excluded from further analysis as these farms did not take part in the core NFS survey. The other five respondents were excluded from the analysis due to missing data in relation to key outcome variables. Respondents were divided into adopters and non-adopters of each management practice to determine the association between the implementation of management practices (listed in Table 1) and gross margin (GM) (€/ewe) and compared using individual sample t-tests.

Parametric and non-parametric tests were used to analyse the questionnaire data. Statistical analysis was carried out using STATA v.13.1 (STATACORP, College Station, TX, USA). Key outcome variables (lamb mortality, number of lambs reared per ewe joined, and gross margin per ewe) were tested for normality using a Shapiro–Wilk test [31] and Kolmogorov–Smirnov test [32] and studied visually using kernel density graphs. Lamb mortality did not follow a normal distribution (Shapiro–Wilk statistic = 0.935, *p* < 0.01) and (Kolmogorov–Smirnov test, *p* < 0.05).

In contrast, the number of lambs reared per ewe joined was normally distributed (Shapiro–Wilk statistic = 0.993, *p* > 0.1). The Shapiro–Wilk test rejects the normality of the gross margin per ewe variable (Shapiro–Wilk statistic = 0.97, *p* < 0.01). However, using kernel density graphs, the distribution of this variable appears to closely resemble a normal distribution. Kolmogorov–Smirnov tests indicate that the data closely follows a normal distribution (Kolmogorov–Smirnov statistic = 0.97; *p* > 0.1). Parametric methods were therefore used to analyse the gross margin and number of lambs reared per ewe joined variables, while non-parametric methods were used to analyse the lamb mortality variable. Independent sample t-tests were used to compare the gross margin and number of lambs reared per ewe joined for adopters and non-adopters of various management practices. The lamb mortality data were analyzed using moods median, Mann–Whitney, and Kruskal–Wallis (KW) non-parametric tests.

## 3. Results

### 3.1. Farm Profile

Farm size, ewe flock size, and productivity data of the farms surveyed are presented in Table 2. The mean flock size and number of lambs reared per ewe joined for lowland and hill flocks were 134.3 (20–1427) and 143.2 (51–580) ewes, and 1.32 (0.6–2.01) and 0.91 (0.36–1.47), respectively. The average age of farmers was 57 years (min 30; max 80). On lowland farms, there was little difference in the average age for farmers who recorded (56.1 years) and did not record (57.1 years) lamb mortality. A total of 44% of respondents were full-time farmers, and the remainder had off-farm employment. The majority of farms surveyed were lowland farms (85%), only farming sheep, although a range of farming systems was represented (Table 3).

### 3.2. Lamb Mortality

Lamb mortality on each farm recorded in the TNFS is calculated based on recorded numbers of live lamb births, and the number of live lambs reared. The mean calculated percent mortality (from birth to weaning) was 7.9% (lowland = 7.5%, *n* = 148; hill = 9.5%, *n* = 24).

Farms in the TNFS supplementary survey were asked whether they formally recorded lamb mortality. A total of 33% of respondents (*n* = 58) recorded lamb mortality in the previous lambing season and categorized the time of death; 49% of respondents did not record lamb mortality but the estimated time of death, while 18% of respondents did not record lamb mortality or time of death. There was no significant difference in calculated lamb mortality between the three groups (Kruskal–Wallis (KW) = 1.078; *p* = 0.5834) and (moods median (MM) = 0.861; *p* = 0.65)).

The main reasons for the mortality of those lambs that were born alive, as perceived by respondents on their farm, are presented in Table 4. The respondents ranked predators, birth weight, and diseases as the main reasons for lamb mortality on their farms. Of the 89% of respondents who provided a second cause of lamb mortality, weather, ewe behavior, and predators were identified as the second cause of lamb mortality on their farm. Of the 69% of respondents who provided a third cause of lamb mortality, accidents, predators and weather were identified as the third cause of lamb mortality on their farm.

### 3.3. Flock Pregnancy Management

Ewes were housed for lambing on 75% of lowland farms. Ewes were lambed indoors, outdoors, and a combination of indoors and outdoors on 31%, 36%, and 33% of hill farms, respectively. When housed, ewes were on straw on the majority of lowland (86%) and hill (93%) sheep farms. The remaining farmers who housed sheep used slatted sheds. Ewes were sheared when housed on 5% and 2% of lowland and hill farms, respectively.

The median percentage of ewes requiring assistance at lambing on lowland farms is presented in Table 5. Farms were categorized as follows: (1) predominantly lambing indoors, (2) predominantly lambing outdoors (3) a combination of indoor and outdoor lambing. Farms were categorized in category 1 or 2, where the proportion of ewes lambing either indoors or outdoors is above 80%. The median level of assistance at lambing was 15% and 10% for ewes lambed indoors and outdoors, respectively. Where farmers lambed some ewes indoors and some outdoor, the median level of assistance at lambing was 7.5%. Ewes lambing indoors were more likely to be assisted than those lambing outdoors or in a combination (KW 16.315; *p* < 0.001), and only indoor farms assisted more than 20% of ewes (Table 5). When including the 24 hill farms, the Kruskal–Wallis test statistic remained significant (KW = 11.504, *p* = 0.003). Of the 24 hill farms, 8, 9, and 7 lambed predominantly indoors, predominantly outdoors, and a combination of both outdoors and indoors, respectively.

Of the 60% of farmers who applied raddle paint to the ram’s chest at joining, the majority (62%) changed the raddle color every 14 days. Other strategies were to change by ewe estrus cycle (17 to 21 days: 17%), every 30 days (8%), and every 7 days (4%). A total of 9% of farmers did not raddle a second time or change color after the first application. When asked for the main reason for changing the raddle color, 14% did not provide a reason. Of those respondents giving a reason, 95% said it was to know the expected lambing date and/or to monitor ram fertility (on their own or in a combination).

### 3.4. Flock Health Management

Ultrasonic pregnancy scanning was undertaken on 69% of respondent farms to identify expected litter size. The mean scanned litter size for all flocks was 1.65 (range 1.1–2.2) (lowland = 1.69, hill = 1.45) lambs per ewe joined. The incidence of a mean flock litter size of ≤1, 1, 2, and ≥3 lambs in flocks was 5%, 33%, 54%, and 8%, respectively.

A total of 86% of respondents vaccinated their ewes before lambing. A total of 97% of farmers vaccinated their ewes against clostridial diseases (on their own or in combination with other vaccines). Clostridial diseases were the only diseases vaccinated against by 42.5% of respondents, while 39.3% used a combined vaccine against clostridial diseases and pasteurellosis diseases. A total of 7% of respondents were vaccinated for both clostridia and pasteurella diseases using separate vaccines.

The availability and use of lambing equipment on respondents’ farms are presented in Table 6. The most common items of lambing equipment available on farm were stomach tubes, hot box/heat lamp, milk feeding equipment, hospital pens, and a supply of colostrum. Most of the farmers (>86%) that had these items of lambing equipment used them during the last lambing season. Thermometers and doxapram were the items least available on farms, and where available, they were the least used.

Post lambing, ewes and their lambs were placed in individual pens on 88% of farms. The mean occupancy time for ewes that had singles, twins, and triplets was 1.1, 2.7, and 3.8 days, respectively. On 41%, 20%, 13%, and 26% of farms, individual pens were cleaned and disinfected (41% of responses) after use; cleaned only (20%); disinfected only (13%); and neither cleaned nor disinfected (26%) between occupants.

Management practices undertaken on lambs within 2 months of birth are presented in Table 7. Application of iodine to lambs’ navels post lambing and tail docking were the most commonly used management practices for lambs. Vaccination for pasteurellosis, orf, and clostridial diseases was the least frequently used management practice. Although most farms (78.8%) tail docked at least some lambs, male lambs were not castrated on 67.8% of farms.

To ensure lambs received adequate colostrum, 73% of respondents assisted some lambs to suckle their dam (on its own or in combination with another method of administering colostrum). Dam’s colostrum was administered to lambs by stomach tube by 44% of respondents. While 64% of respondents used artificial colostrum in combination with other sources, it was the sole source of supplementary colostrum on 10% of farms. Colostrum substitute consisted of colostrum from another recently lambed ewe (44% of farms), frozen/thawed colostrum from another ewe (29%), or frozen/thawed cow colostrum (44%). A total of 81% of respondents used a combination of two or more sources to ensure lambs had adequate colostrum intake.

A total of 76% of respondents fostered lambs. Wet fostering (61%) was the most commonly used method (on its own or in combination with another method), followed by dry fostering (60%) and hide removal (10%). For farms that only used a single fostering method, these were wet fostering (29%), dry fostering (27%), and hide removal (1%). A combination of wet and dry fostering was undertaken by 28% of the respondents. A total of 6% of farmers practiced all three methods; 6% used wet fostering and hide removal, while the remaining 3% used dry fostering and hide removal. Respondents perceived that each of the three techniques had a similar success rate (86%, 84%, and 80% for hide removal, wet fostering, and dry fostering, respectively).

A total of 92% of respondents treated lambs for internal parasites during the previous season. Of these, 56% administered the first anthelmintic to lambs between 5 and 8 weeks of age, 22% between 9 and 12 weeks, 11% before 4 weeks, and 3% after 12 weeks of age.

A total of 12% of respondents took fecal samples to quantify lamb internal parasite burden. These were collected at 8 weeks (26%), 12 weeks (31%), 14 weeks (13%), 16 weeks (4%), or greater than 16 weeks of age (9%).

### 3.5. Association with Productivity and Profitability

Full-time sheep farmers tended to have a higher GM (€84/ewe) than part-time farmers (€71/ewe), (S.E. = 7.6; *p* = 0.09; df = 1). Full-time farmers who used individual and hospital pens had higher GM (€89/ewe) than any farmers who did not use individual/hospital pens (€71/ewe), (S.E. = 8.2; *p* = 0.03; df = 1). Farms that used stomach tubes to administer colostrum had higher GM (€81/ewe) than those that did not use them (€56/ewe), (S.E. = 9.4; *p* = 0.01). The gross margin is significantly higher on lowland farms than hill farms by €37 per ewe (Kruskal–Wallis (KW) = 4.056; *p* < 0.001).

Farms that used individual lambing pens reared more lambs/ewe joined (1.39 lambs/ewe) than farms that did not use them (1.26 lambs/ewe) (S.E. = 0.056; *p* = 0.02). Full-time farmers who used individual and hospital pens had higher ewe productivity (1.44 lambs reared/ewe joined) than farmers who did not use individual and hospital pens (1.34 lambs reared/ewe joined) (S.E. = 0.048; *p* = 0.003).

Lamb mortality tended to be lower (KW = 3.014; *p* = 0.08) on farms that raddled rams relative to the farms that did not use a raddle during the joining period. Lamb mortality tended to be lower (KW = 2.749; *p* = 0.09) on farms that used stomach tubing, heat box, iodine, hospital, and individual pens compared with farms that do not implement all those practices. Farmers who used stored colostrum had lower lamb mortality than those who did not use stored colostrum (KW = 5.026; *p* = 0.025). All other variables were tested, and no significant interaction was found for productivity or profitability (*p* > 0.05).

## 4. Discussion

Farms with below-average ewe productivity in the current study had above-average levels of lamb mortality. Lamb mortality in the current study was recorded as live-born lambs that died prior to weaning (at approximately 14 weeks). The mean number of lambs reared per ewe joined in the current study was 1.37. This is similar to that reported by [33] in the previous National Farm Survey (NFS, 2016). Nationally the average number of lambs reared per ewe joined has remained relatively similar for the past 30 years [7]. The lower lamb mortality in lowland than hill flocks may be linked to more lowland ewes being housed, facilitating timely intervention around parturition. As a result, the lower level of lamb mortality is a contributing factor in the higher gross margins on lowland farms compared to hill farms. Lamb mortality is usually lower in flocks lambing indoors [9,15] and the data recorded in the current study are similar to the mortality percentages recorded for similar types of production systems [11,15,19].

Approximately 50% of lamb mortality occurs prior to birth or during the birth process [15,23], and 75% up to 72 h after birth [23,34]. As the majority of the farmers in the current study lambed indoors and the ewes and their lambs were not turned out to pasture until a few days post partum, it is unlikely that predators would be the main cause of lamb mortality even though farmers believed they were. However, farmers tend to choose issues/factors that they have no control over (e.g., predators and weather) rather than those that they do (e.g., birth weight, disease), which is similar to a recent study in Australia where farmers overestimated predation at three times more likely to be the primary cause of mortality than the published data [35].

Lamb birth weight, ranked as the second most common perceived cause of lamb death, is a key factor influencing neonatal lamb mortality [28,36,37,38,39] as heavy lambs are prone to dystocia while light lambs are more vulnerable to hypothermia and exposure due to the high surface area per unit of body weight. Hanrahan and Keady [28] concluded that the optimum BW of lambs born as twins and triplets was 0.93% and 0.78% of singles, respectively. The use of raddle marking paint on rams at joining and regularly changing the color during the joining period and ultrasonic pregnancy scanning facilitates accurate nutritional management of ewes during late pregnancy according to expected lambing date and litter size. Farmers that raddled the rams during the joining period had lower lamb mortality. While 50% of respondents changed the raddle color to know the expected lambing date, only 4% changed the color every 7 days, which would enable a more accurate assessment of the lambing date. The small proportion of producers who changed raddle color weekly may be due to, firstly, a lack of knowledge of the benefits of knowing expected lambing date when developing a nutrition plan for the late pregnancy period, and secondly, many producers have off-farm employment, which limits opportunities to raddle rams due to short daylight hours during the joining period.

Ultrasonic pregnancy scanning enables farmers to predict expected litter size and manage nutrition accordingly to optimize fetal growth [40]. Scanning was undertaken by 69% of respondents, similar to the proportion of U.K. farmers that scanned their ewes (68%) [11]. During the last six weeks of pregnancy, the energy requirements of ewes carrying singles, twins, and triplets increases by 40%, 60%, and 70%, respectively [2]. If ewes are offered excessive energy intakes above requirement for a protracted period during mid and late pregnancy, the body weight of lambs at birth will be increased. At parturition, dystocia, difficult or abnormal deliveries caused by oversized lambs, is an important cause of lamb mortality, regardless of litter size [28,41]. If the birth weight falls below the optimum, an increase in lamb mortality regardless of litter size is also observed [16,28], primarily due to hypothermia or exposure. From birth until one week of age, hypothermia/starvation are the main causes of lamb mortality in lambs with low birth weight [42].

Farmers perceived diseases and infections to be the third most common cause of lamb mortality on their farms. The navel provides an avenue for infection into the newborn lamb [43], which can be restricted by the use of disinfectants such as iodine. Incidence of lamb mortality due to infection can be reduced by the increased use of common hygiene procedures at or around lambing and better colostrum management. The application of iodine to the navel of lambs was much lower for the current study (79%), compared to that (97%) reported by [11]. A high proportion of farmers in intensive systems in Ireland lamb indoors, 75% from the current study and 83% in a previous study [44], and put ewes and their lambs in individual lambing pens post partum [11]. The use of best management practices around lambing pen hygiene is essential. Binns et al. [11] reported that not applying clean bedding to individual lambing pens daily was the second-highest risk factor in perinatal lamb mortality in a U.K. study. In the current study, only 41% of respondents cleaned and disinfected individual lambing pens after each occupant, thus increasing the risk of infection to neonatal lambs.

Newborn lambs are hypogammaglobulinemic and must consume colostrum as a source of immunoglobulin-G (IgG) soon after birth and for the first few days of the neonatal period to ensure passive immunity [45]. Intake of adequate quantities of colostrum through sucking the dam, or manually administered soon after birth, and suitable hygiene are two management practices that significantly increase the lambs chance of survival and reduce the risk of infection from disease, both neonatal and postnatal [11,45,46], as many neonatal diseases are associated with inadequate serum IgG absorption [47]. The use of stored colostrum in the current study resulted in lower levels of lamb mortality, identifying the benefits of ensuring not only the quantity but the quality and source of colostrum is important. A recent study [48] reported that lambs who received artificial colostrum as a substitute for ewes colostrum had increased diagnosis of scours between weeks 2 and 5 and required more antibiotic treatments than lambs that received ewes colostrum. While 64% of respondents in the current survey used artificial colostrum on some lambs, it was the sole source of supplementary colostrum on 10% of farms, which may be an area to address in attempts to reduce on-farm lamb mortality. Three-quarters of farmers in the current study ensured lambs received mother’s colostrum, which is similar to previous studies [11]. Getting newborn lambs to their feet and adequate colostrum intake are the major factors in increasing the chance of survival [49,50]. When lambs are unsuccessful at getting to the udder in the first few hours post partition, a stomach tube may be used to deliver adequate colostrum. The majority of farmers (87%) had stomach tube equipment available on farm, and most of them (90%) used it in the previous lambing season. When used correctly and suitable hygiene practices are implemented, the risk of neonatal lamb mortality is reduced; however, an added risk of infection is associated with the use of stomach tubes. A large proportion of sheep in Ireland are housed for lambing. Housing greatly increases the risk of infection, which is a significant cause of neonatal lamb mortality in indoor lambing flocks [11,15,19,23]. While housing sheep prior to lambing increases the risk of infection, due to higher stocking rate and poor hygiene, it reduces the risk of some of the causes of lamb mortality, e.g., predation hypothermia/starvation and mis-mothering. This was evident in the current study as the use of individual lambing pens decreased the level of lamb mortality.

It is difficult for producers to assess the causes of lamb mortality on their farms if they do not record lamb mortality and the perceived cause. While lamb mortality is a key factor influencing ewe productivity, thus flock profitability, most farmers in this study did not record lamb mortality. A recent survey study in Australia of sheep farmers’ perceptions of lamb mortality reported that producers may be largely underestimating their mortality incidence compared to experimental studies of similar breeds and using similar management practices [35]. However, in the current study, when asked to provide the number of lambs that died on the farm, there was little difference in numbers between the farmers who recorded lamb mortality and those who did not. Several reasons could explain the similarities in the percentages from both subsets of farmers. Firstly, flocks in the current survey were relatively small (average = 131 ewes) compared to similar systems in other countries (e.g., Australia), where average flock sizes can be over 4000 ewes [32]. Small flocks, particularly if housed [11], enable more supervision per ewe, which can result in relatively low levels of lamb mortality and facilitate accurate estimation of time of death. Secondly, it is possible that the results could be under or overestimated by either subgroup of respondents. Binns [11] also reported that total lamb mortality reported by farmers themselves was on average 3% lower than that reported by a sampler.

Older farmers are less inclined to adopt new strategies [51], new technologies, or change their management practices until they have been proven to work. However, the rate of respondents who recorded lamb mortality was not associated with the average age of farmers in the current study. Nearly half of farmers in the study (44%) have an off-farm job, and labor availability during the lambing season may be limited to certain times during the day, which can impact accurate recording at lambing.

The high response rate in this survey (97%) is similar to the response achieved in other Irish agricultural-based surveys undertaken by Bohan [44] and Hession [52]. This high response rate can be associated with a number of different factors that include the piloting of the survey and the use of predominantly closed questions, and especially having a trained survey recorder assisting with the completion of each survey on a one to one basis and the integration of the supplementary survey within the long-established Teagasc National Farm Survey architecture.

## 5. Conclusions

Predators are considered the main cause of live-born lamb mortality based on farmers’ own perception; however, similar to previous studies, these are deemed to be overestimated. While lamb mortality is not routinely recorded on most Irish sheep farms, farmers who recorded lamb mortality had lower levels of mortality on their farm compared with farmers who did not record lamb mortality, highlighting the benefits of implementing an accurate recording system on each farm. Farmers who implement perceived positive neonatal management practices, stomach tubing, heat box, iodine, use of hospital and individual lambing pens, had lower lamb mortality. These findings could be used to design pre-lambing checklists of required lambing equipment and disseminated to all farmers. Full-time farmers who used lambing pens around lambing weaned an extra 0.10 lambs per ewe and received, on average, €18/ewe more than farmers who did not use individual lambing pens. Knowledge transfer activities need to be designed to communicate these best practices with regards to neonatal lamb management, in particular identifying the named risk factors and the most appropriate farm management strategies to minimize these risks.

## Figures and Tables

**Table 1:** Summary of the information sought in the survey.

Management Practice	Information Sought
**Lamb mortality recording**	
Recording mortality	Recorded (yes/no)
	Cause of death
	Time of death (abortion, stillbirth, died 0–1 day, 2–7 days, 7–100 days)
**Causes of lamb mortality**	
Farmers’ perspective of 3 main causes on their farm	3 main causes (in order of importance) from the following 12 options: birth weight, ewe behavior, lamb behavior, accidents, predators, weather, mineral deficiency, ewe body condition, internal parasites, hygiene, clostridial diseases, disease, e.g., -coli, joint ill, etc.
**Management—joining to late pregnancy**	
Raddle application	Yes/no
	How often was color changed
	Why apply raddle
Pregnancy scan	Yes/no
	Number of each litter category
Vaccination program	Yes/no for toxoplasmosis, enzootic abortion, clostridia, and/or pasteurellosis
**Lambing equipment available**	
Preparation for lambing	Was the following equipment available—thermometer, hot box/heat lamp, stored colostrum, stomach tubing equipment, hospital pens, Doxapram, milk feeding equipment. If available, was it used during the previous lambing season?
**Lambing practices**	
Assistance	Percentage of ewes assisted
Individual pens	Used post lambing (yes/no)
Hygiene of individual pens	How long were ewes in each litter category resident (days) when each ewe and her lambs exited, were the pens cleaned only, disinfected only, cleaned and disinfected, not cleaned or disinfected
Cross fostering Fostering technique	Did you transfer any lamb from its biological dam to a surrogate dam to rear it Did you use any of the following techniques to cross foster lambs: Wet fostering—covering the orphan lamb with amniotic fluids from the surrogate mother Dry fostering—either restraining the surrogate dam in a fostering gate (head restrained and ewe cannot see or smell the orphan lamb/s) or removing the hide from ewes own lamb and placing it on the lamb to be adopted
**Lamb treatments**	
Iodine applied to navel	Yes/no
Orf vaccine used	Yes/no
Antibiotic administered (e.g., joint ill, scour)	Yes/no
Tail docked via elastrator band	Yes/no
Males castrated	Yes/no
Clostridia and/or pasteurellosis vaccination	Yes/no
**Colostrum**	
Type used and method of administration	Stomach tube mother’s colostrum
	Frozen/thawed ewe and/or cow colostrum
	Colostrum from another ewe
	Artificial colostrum
	Assist to suck
**Lambs at pasture**	
Internal parasite control	Age at first anthelmintic treatment for internal parasites
Fecal egg counts	were fecal egg counts undertaken and at what age

**Table 2:** Range in farm and flock performance (*n* = 177).

Descriptor	Mean	Minimum	Maximum
Farm size (ha)	62	7.7	330.1
Sheep forage area ^1^ (ha)	20.8	1	158.4
Average number of ewes	131	20	1427
Stocking rate (ewes/ha)	7.2	0.3	16.2
Lambs reared/ewe joined	1.3	0.41	2.21
**Flock Size (No of Ewes)**	**% of Respondents**		
≤50	32		
51 to 100	34		
101 to 150	16		
>150	18		

^1^ The total adjusted area under grass (including rough grazing) plus adjusted commonage area (share of unenclosed lands) for sheep enterprise.

**Table 3:** Details of farm classification by main enterprise and sheep system (*n* = 177).

Teagasc NFS Farm Classification	% of Farms
Mainly sheep	57
Cattle	27
Dairying	7
Tillage	4
Other	3
Sheep-only system	
Lowland	85
Hill	14
Other	1

**Table 4:** Farmers’ perceptions of the main cause for live lamb mortality on their farms (%).

	Ranking by Respondents		
Rank of Cause of Lamb Mortality	First ^1^	Second ^2^	Third ^3^
Primary	Predators (21%)	Weather (21%)	Accidents (16%)
Secondary	Birth weight (18%)	Ewe behavior (18%)	Predators (16%)
Tertiary	Diseases (16%)	Predators (16%)	Weather (15%)
Respondents (%)	98	89	69

^1^ Farmers’ perceived predators, birthweight, and disease to be the most common cause of lamb mortality; ^2^ Weather, ewe behavior, and predators were perceived to be the second main cause of lamb mortality; ^3^ Accidents, predators, and weather were perceived to be the third most common causes.

**Table 5:** Proportion of ewes assisted at lambing on lowland farms.

			Proportion of Ewes Assisted		
	No. of Farms	Median (%)	≤10%	10–20%	≤20%
Indoors ^1^	118	15	35.8	34.6	29.6
Outdoors ^2^	14	10	53.8	46.2	0
Combination ^3^	16	7.5	61.3	38.7	0

^1^ Indoors = predominantly lambing indoors (>80% of ewes); ^2^ Outdoors = predominantly lambing outdoors (>80% of ewes); ^3^ Combination = combination of indoor and outdoor lambing.

**Table 6:** Equipment available on farm and used to improve lamb survival (*n* = 177).

Equipment	Respondents with Equipment Available (%)	Respondents Who Used Available Equipment (%)
Stomach tubing equipment	87	90
Hot box/heat lamp	79	86
Milk feeding for artificial rearing of lambs	73	90
Hospital pen	70	89
Supply of stored colostrum	64	90
Thermometers	31	42
Doxapram revival drops ^1^	15	33

^1^ To initiate or stimulate respiration in neonatal lambs following dystocia or difficult lambing.

**Table 7:** Management practices undertaken on live-born lambs within two months of birth (*n* = 177 flocks).

		Percentage of Lambs (%)		
	No. of Respondents	All	Some	None
Navel treated with iodine	175	79.2	8.5	12.4
Tail docked via elastrator band	175	62.7	16.2	21.2
Vaccination against *Clostridia* spp.	146	28	2.5	70
Males castrated	165	24.9	7.3	67.8
Vaccination against Pasteurellosis vaccination	159	19	3.3	77.7
Vaccinated for orf ^1^	162	17	8	75
Treated with antibiotics ^2^	162	9.3	44.4	46.3

^1^ Contagious pustular dermatitis (parapox ovis); ^2^ To treat joint ill or scour or other bacterial infections.

## Data Availability

The data and models reported within this study are available from the corresponding author upon reasonable request.

## Supplementary Materials

The following are available online at https://www.mdpi.com/article/10.3390/ani12010030/s1, file S1: supplementary survey.
