# Supplementary material for: A Survey of Farm Management Practices Relating to the Risk Factors, Prevalence, and Causes of Lamb Mortality in Ireland
Source: Animals (Basel). 2021 Dec 23;12(1):30. doi: 10.3390/ani12010030 (PMC8749728; doi:10.3390/ani12010030)
Supplement: Supplementary file 1 [file animals-12-00030-s001.zip › animals-1468400-supplementary.pdf]

NFS Additional Survey 2017

Agricultural Economics & Farm Surveys Dept.

FARM CODE Rec

Lamb Mortality Survey

Q4a: How many breeding ewes are on this holding?

If the number of ewes is greater than **20 ewes** please completed remainder of the questionnaire

Question 5: Which of the following best describes your flock management:

	% Flock	Housed Y/N	Shorn at housing Y/N	Floor type	Ewes assisted at lambing (%)
Lowland, lambing indoors.					
Lowland, lambing outdoors.					
Hill, lambing indoors.					
Hill, lambing outdoors					

Housing; 1 = Yes, 2 = No

Floor Type: 1 = Straw; 2 = Plastic slats; 3 = Expanded metal slats; 4 = Wooden slats; 5 = Other (Specify)

Question 6: Raddle

Q6a: Did you raddle your rams in 2016? Yes = 1; No = 2;

Q6b: If **Yes**, how often did you change the raddle colour?

1 = Weekly; 2 = Fortnightly; 3 = Monthly); 4 = Other (Specify) ____________________

Q6c: What was the main reason you chose to change the raddle colour

1 = Monitor ram fertility; 2 = Grouping purposes for feeding; 3 = Know expected lambing date; 4 = Other (Specify) ______________________________

Question 7: Scanning

Q7a Scanning: Did you pregnancy scan your ewes in 2017? Yes = 1; No = 2;

Q7b: If **Yes**, How many ewes were scanned?

	Empty	Singles	Twins	Triplets	Quads+
No of ewes					

What was your scan rate?

(No. of lambs /ewe joined to the ram)

Question 8: Vaccination

Please enter code below if you vaccinated your ewes for any of the following in 2016/2017?

1 = Toxoplasmosis (e.g Toxovax)*; 2* = Enzootic abortion/chlamydia (e.g Enzovax)*;*
3 = Clostridial disease (e.g Covexin 8/10 or Heptavac)*; 4 = Pasteurella vaccination (e.g., Heptavac-P, Ovivac-P); 5* = Other (Specify) ___________________________

Q9: Which of the following did you have available on your farm for use at lambing time in 2017? (please tick whether available and also whether used at lambing (✓))

	Available	Used at Lambing
a) Thermometers for recording lamb temperature		
b) Hot box/heat lamps for warming hypothermic lambs		
c) Supply of stored colostrum		
d) Stomach tubing equipment		
e) Hospital pens		
f) Dopram V revival drops/injection		
g) Milk feeding for artificially rearing lambs		

Q10: Did you foster any lambs in 2017? 1 = Yes; 2 = No

	Used (Y/N)	Success Rate (%)
a) If **Yes** did you use dry-fostering? *1 = Yes; 2 = No*		
b) If **Yes** did you use wet -fostering? *1 = Yes; 2 = No*		
c) If **Yes** did you use hide removal? *1 = Yes; 2 = No*		

Question 11: Lamb Deaths

Q11a: Did you record lamb deaths in 2017? Yes = 1; No = 2;

COMPETENCY	Abortions	Stillbirths	Died 0–1 day	Died between 2–7 days	Died subsequently until weaning
No. of Lambs					

Question 12: Lambing Practices

Q12a: Are ewes placed in individual pens after lambing? Yes = 1; No = 2; [ ]

Q12b: If Yes, how long are ewes & lambs in these pens? (enter average no. of days)

	Singles	Twins	Triplets
Average number of days			

Q12c: If yes, are the pens cleaned & disinfected after every ewe vacates (tick one(✓))

	No	Cleaned only	Disinfected only	Cleaned & disinfected
Tick				

Q13: Did your lambs receive any of the following treatments within two months of birth (✓)

	All	Some	None
a) Naval treated with iodine			
b) Treated for orf			
c) Treated with antibiotics (joint ill/scour etc.)			
d) Tail docked via elastor band before 3 days of age			
e) Male lambs castrated			
f) Clostridia vaccination (Heptavac/Covexin 8/10)			
g) Pasturella vaccination (e.g., Heptavac-P/ovivac-P)			

Q 14: Which of the following did you use at lambing time in 2017(✓)

Mother colostrum - lamb stomach tubed		Frozen/thawed sheep colostrum		Frozen/thawed cows colostrum	
Artificial colostrum		Colostrum from another ewe		Assisted lamb to suck	

Q 15: What do you consider to be the top 3 causes of live-born lamb mortality in your flock (**Rank 1 to 3** in order of importance with 1 being the most important)

Birth weight		Ewe behaviour		Lamb behaviour	
Accidents		Predators		Weather	
Mineral deficiency		Ewe body condition		Internal parasites	
Hygiene		Clostridial diseases		Diseases e.g., ecoli; joint ill	

Other: Specify____________________________

Q16 Lambs at Pasture: At what age did you administer your first treatment to your lambs for internal parasites in 2017? *1* = No treatment; *2* = 0–4 weeks; *3* = 5–8 weeks; *4* = 9–12 weeks; *5* = >12 weeks

Q17 Did you take faecal egg count (FEC) in 2017? 1 = Yes; 2 = No

If **Yes** at what age
